# Cervical dilatation over time is a poor predictor of severe adverse birth outcomes: a diagnostic accuracy study

**DOI:** 10.1111/1471-0528.15205

**Published:** 2018-04-17

**Authors:** JP Souza, OT Oladapo, B Fawole, K Mugerwa, R Reis, F Barbosa‐Junior, L Oliveira‐Ciabati, D Alves, AM Gülmezoglu

**Affiliations:** ^1^ UNDP/UNFPA/ UNICEF/WHO/World Bank Special Programme of Research Development and Research Training in Human Reproduction (HRP) Department of Reproductive Health and Research World Health Organization Geneva Switzerland; ^2^ Department of Obstetrics and Gynaecology College of Medicine University of Ibadan Ibadan Nigeria; ^3^ Department of Obstetrics and Gynaecology Makerere University Kampala Uganda; ^4^ Department of Social Medicine Centre for Information and Informatics for Health (CIIS) Ribeirão Preto Medical School University of São Paulo Ribeirão Preto Brazil

**Keywords:** alert line, childbirth, diagnostic accuracy, partograph, receiver operating characteristic space

## Abstract

**Objective:**

To assess the accuracy of the World Health Organization (WHO) partograph alert line and other candidate predictors in the identification of women at risk of developing severe adverse birth outcomes.

**Design:**

A facility‐based, multicentre, prospective cohort study.

**Setting:**

Thirteen maternity hospitals located in Nigeria and Uganda.

**Population:**

A total of 9995 women with spontaneous onset of labour presenting at cervical dilatation of ≤6 cm or undergoing induction of labour.

**Methods:**

Research assistants collected data on sociodemographic, anthropometric, obstetric, and medical characteristics of study participants at hospital admission, multiple assessments during labour, and interventions during labour and childbirth. The alert line and action line, intrapartum monitoring parameters, and customised labour curves were assessed using sensitivity, specificity, positive and negative likelihood ratios, diagnostic odds ratio, and the *J* statistic.

**Outcomes:**

Severe adverse birth outcomes.

**Results:**

The rate of severe adverse birth outcomes was 2.2% (223 women with severe adverse birth outcomes), the rate of augmentation of labour was 35.1% (3506 women), and the caesarean section rate was 13.2% (1323 women). Forty‐nine percent of women in labour crossed the alert line (4163/8489). All reference labour curves had a diagnostic odds ratio ranging from 1.29 to 1.60. The *J* statistic was less than 10% for all reference curves.

**Conclusions:**

Our findings suggest that labour is an extremely variable phenomenon, and the assessment of cervical dilatation over time is a poor predictor of severe adverse birth outcomes. The validity of a partograph alert line based on the ‘one‐centimetre per hour’ rule should be re‐evaluated.

**Funding:**

Bill & Melinda Gates Foundation, United States Agency for International Development (USAID), UNDP/UNFPA/UNICEF/WHO/World Bank Special Programme of Research, Development and Research Training in Human Reproduction (HRP), and WHO (A65879).

**Tweetable abstract:**

The alert line in check: results from a WHO study.

## Tweetable abstract

The alert line in check: results from a WHO study.

## Introduction

Labour and childbirth are natural processes with a relatively low frequency of complications among healthy pregnant women.^1^, ^2^ Intrapartum maternal and fetal monitoring is used to further minimise risks, and is expected to enable the early identification and prompt treatment of complications. The assessment of cervical dilatation is part of intrapartum monitoring, and is conducted by healthcare providers to determine the adequacy of labour progress. The observed cervical dilatation is usually compared with reference labour curves to estimate the risk of labour complications and to guide the use of interventions.^3^, ^4^, ^5^, ^6^, ^7^

The World Health Organization (WHO) partograph is a decision‐making support tool designed to assist health providers in identifying women at risk of developing complications during labour, and to guide the use of interventions intended to mitigate any perceived risks.^6^ With the partograph, the identification of certain patterns of cervical dilatation or other risk factors may prompt the transfer of the woman to a higher‐level health facility, the intensification of intrapartum monitoring, the augmentation of labour, or delivery by caesarean section.^7^ The WHO partograph is based on clinical principles, including the notion that ‘normal’ labour progress is defined by a cervical dilatation rate of not less than one centimetre per hour between 4 and 10 centimetres of cervical dilatation.^6^ This concept is the basis for the partograph ‘alert line’, which was derived from average labour curves developed during the 1950s and 1960s.^8^, ^9^ Alternative and more recent labour curves have been developed to provide a reference for labour progress and to serve as the basis for new partograph designs.^5^, ^10^

Although some observational studies and other empirical evidence point towards the benefit of using the WHO partograph, experimental, head‐to‐head comparisons failed to demonstrate an effect of the partograph in improving health outcomes related to labour and childbirth.^11^ Furthermore, different studies have pointed to limitations in the ‘one centimetre per hour rule’ as a valid benchmark for assessing the adequacy of labour progress.^5^, ^10^, ^12^ Our hypothesis is that if the partograph is unable to accurately identify women at risk of developing intrapartum complications, it will not be able to effectively guide labour management.

This article reports on findings of the WHO Better Outcomes in Labour Difficulty (BOLD) project. The present analysis assessed the diagnostic accuracy of the alert line, action line, and other parameters included in the WHO partograph as predictors of severe adverse birth outcomes. It also assessed the accuracy of customised labour curves to identify women at risk of developing severe adverse birth outcomes.

## Methods

The BOLD project included quantitative, qualitative, and service‐design research conducted in Nigeria and Uganda. The methodological details of the BOLD project have been described elsewhere.^13^, ^14^ This analysis is based on the quantitative component, a facility‐based, multicentre, prospective cohort study. In brief, this study included women admitted for vaginal birth with single live fetuses during the early first stage of labour across 13 hospitals in both countries. Women with spontaneous onset of labour presenting at cervical dilatation of ≤6 cm and women undergoing induction of labour took part in the study. Women with multiple pregnancies, women with pregnancies with gestational ages of less than 34 weeks 0 days, women choosing elective caesarean section, and women who were incapable of giving consent because of labour distress or obstetric emergencies at arrival were excluded. Participating institutions had a minimum of 1000 deliveries per year, with stable access to caesarean section, augmentation of labour, and assisted vaginal birth. Midwives, obstetricians, or obstetric residents provided intrapartum health care to women in labour. Doptones were used to assess fetal vital status at hospital admission and for intermittent monitoring through labour and childbirth. Labour management protocol, as well as the number and timing of pelvic examinations, were not standardised across participating institutions. None of the institutions subscribed to the active management of labour protocol during the study period. Although the partograph was a standard element of medical records in all participating health facilities, its prospective application to guide labour management during the study period varied widely across the hospitals.

Eligible women were recruited into the study between December 2014 and November 2015. From the medical records, trained research nurses prospectively extracted detailed information on the sociodemographic, anthropometric, obstetric, and medical characteristics of the study participants at hospital admission, multiple assessments during labour monitoring, interventions performed throughout the first and second stages of labour, and maternal and neonatal labour outcomes. Attending staff were approached to complement medical records data when needed. Data collection was limited to hospital stay of the mother and baby, and there was no post‐hospital discharge follow‐up.

The current analysis was based on information on maternal baseline and admission characteristics, repeated assessments of cervical dilatation versus time, and maternal and neonatal outcome data. Severe adverse birth outcomes were defined as the occurrence of any of the following: stillbirths, intra‐hospital early neonatal deaths, neonatal use of anticonvulsants, neonatal cardiopulmonary resuscitation, Apgar score of <6 at 5 minutes, uterine rupture, and maternal death or organ dysfunction with dystocia. Details of the sample size calculation are provided in the supporting information (Box S1).

### Data analysis

Simple frequencies and proportions were used to describe the characteristics of the study population. Sensitivity, specificity, positive and negative likelihood ratios, diagnostic odds ratios, and the *J* statistic (Youden's index), with 95% confidence intervals, were used to estimate the diagnostic accuracy of the alert line and the action line in the identification of women who would develop a severe adverse birth outcome.^15^, ^16^, ^17^, ^18^ We used the true‐positive rate (i.e. sensitivity) and the false‐positive rate (i.e. 1 – specificity) to graphically represent the diagnostic accuracy of the partograph parameters in the receiver operating characteristic (ROC) space.^19^ Each point estimate in the ROC space represents a classification result for binary parameters, and the interpretation of the ROC space is similar to the ROC curve: optimal results are associated with high true‐positive rates combined with low false‐positive rates. The *J* statistic summarises the performance of a binary classifier,^16^ and also expresses the proportion of ideal performance of a diagnostic test (Box S2). The supporting information provides additional details related to the calculation and interpretation of these statistics (Tables S1–S3).

The alert line and the action line are classifiers currently applied to all women, regardless of their obstetric characteristics (e.g. nulliparous, multiparous, spontaneous or induced labour, or previous caesarean section). We hypothesised that cervical dilatation curves customised according to the obstetric characteristics of the population could have a better accuracy than the generic alert and action lines. The study population was stratified into mutually exclusive, totally inclusive obstetric groups according to the 10‐group Robson classification:^20^ group 1 (nulliparous, single cephalic pregnancy, 37 weeks of gestation or more, with spontaneous onset of labour); group 2 (nulliparous women, single cephalic pregnancy, 37 weeks of gestation or more, with induced onset of labour); group 3 (multiparous women without previous caesarean section, with single cephalic pregnancy, 37 weeks of gestation or more, with spontaneous onset of labour); group 4 (multiparous women without previous caesarean section, with single cephalic pregnancy, 37 weeks of gestation or more, with induced onset of labour); group 5 (all multiparous women with at least one previous caesarean section, single cephalic pregnancy, at 37 weeks of gestation or more); and group 10 (all women with singleton cephalic preterm pregnancy at less than 37 weeks of gestation at childbirth). As a result of the eligibility criteria, this study has no women from group 8 (multiple pregnancies) or with caesarean section before labour. Women with non‐cephalic presentations (groups 6, 7, and 9) were grouped together. Groups 1–5 and 10, were further divided according to the use of augmentation of labour (present or absent), totalling 12 subgroups. Using data from women who did not have any severe adverse birth outcome, customised labour curves were generated for each of these 12 subgroups. Data from women pertaining to groups 6, 7, and 9 were not used to generate customised curves because of the small numbers involved. The customised cervical dilatation curves were created using a multi‐state Markov model,^21^, ^22^ which represented the cervical dilation pattern through intermediate states from 2 cm to 10 cm, and childbirth by selected percentiles and obstetric group (i.e. one labour curve for each obstetric group and selected percentile). In this model, each centimetre of cervical dilatation represented an intermediate state, and childbirth was the final ‘absorbing’ state. The model was generated as a progressive unidirectional labour‐to‐childbirth model, and the time of state change was determined by a set of transition intensities. The transition intensity represents the instantaneous likelihood of moving from one state to another, and is generated as part of the multi‐state Markov model. For each one of the 12 obstetric subgroups, the multi‐state Markov model generated labour curves representing the progress of labour in women that was either faster or at the 50, 60, 70, 80, 90, and 95^th^ percentiles.

Once the percentile curves were generated for each obstetric subgroup of women without severe adverse birth outcomes, women were classified as having crossed or not having crossed each of the percentile curves of their relevant obstetric subgroup. The study population was then consolidated and all women who crossed their relevant 50^th^ percentile curves were grouped together (i.e. women in which labour progressed more slowly than the customised 50^th^ percentile curve). Similarly, women were classified as having labour that progressed either slower or faster/equal to the relevant 60, 70, 80, 90, and 95^th^ percentiles. We estimated the accuracies of the customised percentile curves in the identification of women who would develop a severe adverse birth outcome, by comparing women with labour progress that was slower than the specific percentile with women in which labour progressed faster or equal to that percentile. Sensitivity, specificity, positive and negative likelihood ratios, diagnostic odds ratios, with 95% confidence intervals, the *J* statistic, and ROC space plotting were used to estimate the accuracy of the percentile curves in the identification of women who would develop a severe adverse birth outcome.

Statistical analyses were carried in r and Microsoft excel (2010).^23^

## Results

**Figure 1:**
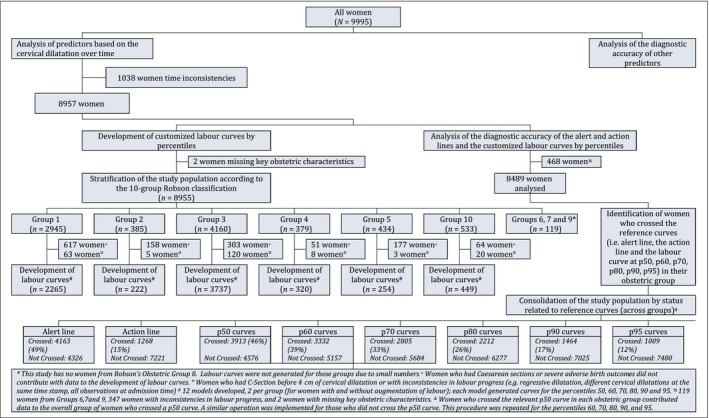
The analysis flowchart.

**Figure 2:**
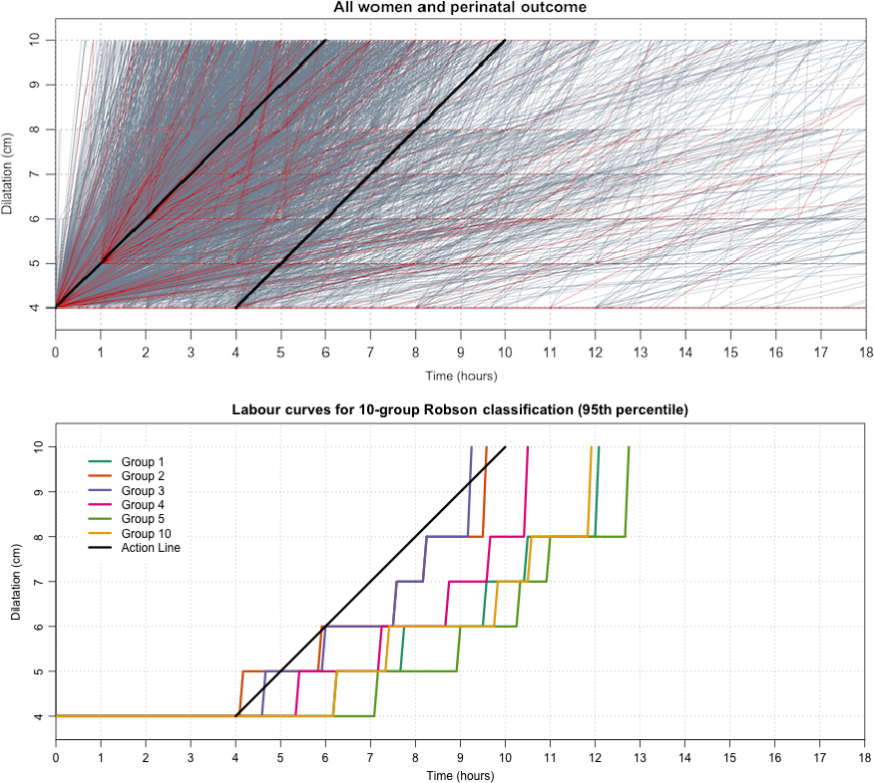
Upper panel: cervical dilatation over time (all women with at least two cervical dilatation assessments between 4 cm and childbirth). Grey lines denote labour progress of women without severe adverse birth outcomes; red lines denote labour progress of women with severe adverse birth outcomes. Lower panel: labour curves for selected groups of the 10‐group Robson classification (95^th^ percentile, women without augmentation of labour).

The analysis flow is shown in Figure 1. Thirteen hospitals (nine from Nigeria and four from Uganda) and a total of 9995 women (4964 from Nigeria and 5031 from Uganda) took part in this study. The average age of the participants was 27.9 years (±5.0 years); 3.2% were younger than 20 years of age (320 women) and 11.0% were 35 years old or older (1100 women). The majority of the participants had a partner (97.6%, 9753 women); 5.2% of the participants had either incomplete primary education or no education (525 women), 5.6% had complete primary education (564 women), 42.5% had either complete or incomplete secondary education (4245 women), and 45.4% had either complete or incomplete post‐secondary/tertiary education (4537 women). A total of 4076 nulliparous women took part in the study (40.8%), and among women with at least one previous birth (59.2%, 5919 women), 535 (5.4%) had had a previous caesarean section. A total of 667 women (6.7%) had no antenatal care visit, 4229 (42.3%) had between one and three antenatal care visits, and 5007 (50.6%) had four visits of more; 1228 women (12.3%) developed pre‐labour complications during the current pregnancy. The majority of women initiated labour spontaneously (8984 women, 89.9%) at between 37 and 41 weeks of gestation (91.2%, 9111 women), with only 594 (5.9%) being referred from another health facility during labour. All women participating in this study had singleton pregnancies, 98.6% (9845 women) of which were in cephalic presentation. The mean number of cervical assessments between 4 and 10 cm was 2.22 (±1.02). Augmentation of labour was used in 3506 women (35.1%). Pharmacological analgesia was rarely used (2.0%, 196 women). Table 1 presents the distribution of the study population according to the 10‐group Robson classification. The overall intrapartum caesarean section rate was 13.2% (1323 women), and the rate of severe adverse birth outcomes was 2.2% (223 women with severe adverse birth outcomes; Table S4). Nearly half of women with at least two assessments of cervical dilatation between 4 cm and childbirth crossed the alert line (49%; 4163/8489). Figure 2 illustrates the progress of labour in the study population. In the upper panel, each grey line represents the progress of an individual women without severe adverse birth outcomes, and each red line represents the progress of an individual woman with adverse outcomes. In the lower panel, the labour curves for women in the 95^th^ percentile, without augmentation of labour, is displayed by obstetric group. Video S1 displays an animation of labour progress of all women in labour that reached at least 4 cm of cervical dilatation.

**Table 1:** Diagnostic accuracy of the alert line and the action line for severe adverse birth outcomes (*n *= 8603)

Clinical signs		Severe adverse birth outcomes		Sensitivity (95% CI)	Specificity (95% CI)	Positive likelihood ratio (95% CI)	Negative likelihood ratio (95% CI)	Diagnostic odds ratio (95% CI)	*J* statistic (Youden's index) (95% CI)
		Present	Absent						
Alert line	Crossed	110	4053	56.7% (49.7–63.5)	51.1% (50.1–52.2%)	1.16 (1.02–1.32)	0.85 (0.72–1.00)	1.37 (1.03–1.83)	7.8% (0.8–14.9)
	Not crossed	84	4242						
Action line	Crossed	38	1230	19.6% (14.6–25.7)	85.2% (84.4–85.9)	1.32 (0.99–1.77)	0.94 (0.88–1.01)	1.40 (0.98–2.01)	4.8% (–0.9 to 10.4)
	Not crossed	156	7065						

**Figure 3:**
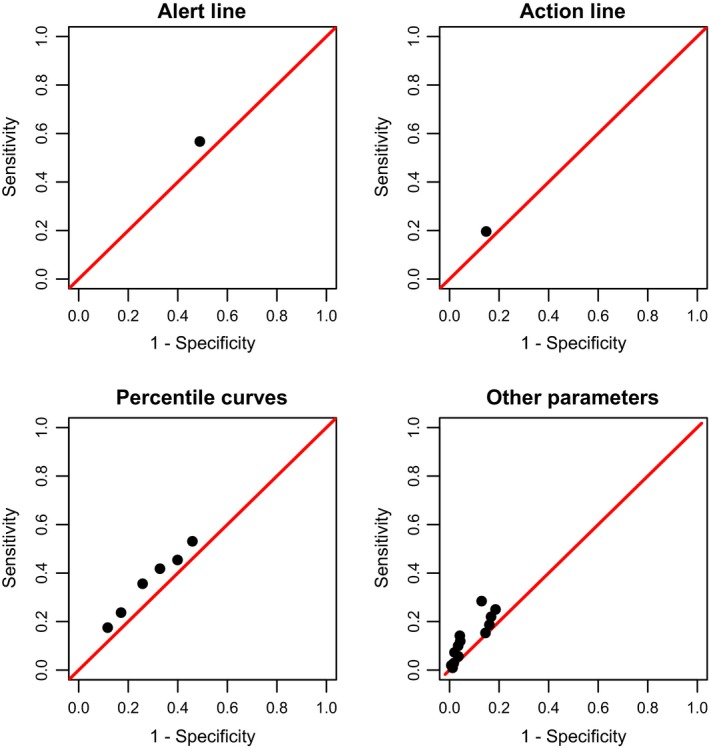
Analysis of the ROC space (alert and action line, customised percentile curves, and other parameters included in the partograph).

The sensitivity, specificity, positive and negative likelihood ratios, diagnostic odds ratio, and *J* statistic for the WHO partograph alert and action lines, and the 50, 60, 70, 80, 90, and 95^th^ percentiles are presented in Tables 1 and S5. For all reference curves, women who crossed the curves tended to show a mild increase in the odds of severe adverse birth outcomes, when compared with women who did not cross the reference lines. All reference curves had a diagnostic odds ratio ranging from 1.29 to 1.60. All reference curves had positive likelihood ratios smaller than 1.5 and negative likelihood ratios greater than 0.85. The *J* statistic was less than 10% for all reference curves. Figure 3 presents the ROC space analysis, with all the aforementioned predictors showing a poor diagnostic performance.

Figure S1 and Table S6 present the diagnostic accuracy of various predictors included in the partograph (the definitions of these predictors are presented in Table S7). Abnormal fetal heart rate, absence of fetal movements, significant moulding, significant caput succedaneum, meconium, and maternal hyperthermia (fever) were associated with mild to moderate increased odds of severe adverse birth outcomes. Similarly to the labour curves, the examined predictors presented poor performance in the prediction of severe adverse birth outcomes.

## Discussion

### Main findings

Labour is an extremely variable phenomenon, and our findings suggest that the assessment of cervical dilatation over time is a poor predictor of severe adverse birth outcomes. Labour curves depicting the cervical dilatation over time (including the WHO partograph alert and action lines) showed poor diagnostic accuracy to identify women at risk of severe adverse birth outcomes during labour. We draw one main inference from these findings: the validity of a partograph alert line based on the ‘one‐centimetre per hour’ rule should be re‐evaluated.

### Strengths and limitations

These findings are relevant to the care provided in health facilities, particularly in sub‐Saharan Africa, and have potential implications for clinical practice. Despite the procedures adopted to ensure appropriate study implementation and high quality data, some limitations need to be considered, however. The primary data source in this study was routine hospital records, complemented by information obtained from clinical staff. We opted for this approach to minimise any interference with the standard practice in health facilities, but acknowledge that it could be associated with irregular and, at times, incomplete and intermittent assessment and recording of maternal and fetal status during labour. Although unlikely given the clinical workload, the availability of Doptones provided by the study in the labour wards may have facilitated fetal monitoring and contributed to an increased identification of fetal distress, which could have affected the clinical management and outcomes. We were also able to determine the fetal vital status at arrival for all women, which resulted in an accurate assessment of intrapartum, intra‐hospital fetal mortality. This assessment enabled the disentangling of pre‐hospital fetal deaths from intra‐hospital fetal deaths, and uncovered a low rate of intra‐hospital fetal mortality, despite the constraints to optimal care in health facilities. None of the participating hospitals subscribed to a systematic implementation of the active management of labour; although this could contribute to a less standardised management of labour, it favoured a less interventionist approach and an intra‐hospital labour progression that was more closely related to the natural progression in many women. Given the differences of workload and health‐facility protocols, the standardisation of intrapartum maternal–fetal monitoring and recording was a challenging task. Several mechanisms were used to minimise methodological heterogeneity and to increase the quality of the data as much as possible (such as research assistant training, the use of a visual check of the data collection forms before data entry, automated queries, double‐checking of selected medical records, and a thorough audit of unclear cases, especially those resulting in mortality). It should also be considered that crossing the alert or action lines could have prompted health providers to implement interventions in the current cohort population. These interventions could have modified the final outcome, either for good or bad.

### Interpretation

Health facilities in low‐resource settings often struggle with a shortage of human resources and life‐saving commodities, training resources, and health infrastructure, which limit the early identification and effective management of labour complications. Conversely, any overestimation of the risk of complications and over‐medicalisation of care during labour and childbirth may lead to iatrogenic complications, avoidable suffering, and a waste of limited resources.^24^ In an attempt to optimise intrapartum care, several organisations recommend the use of the WHO partograph to guide labour monitoring and management.^24^ The ‘one‐centimetre per hour rule’, as illustrated by the partograph alert line, has also (formally or informally) been used to prompt labour interventions in many settings around the world.^4^, ^25^ Global efforts to promote the use of the partograph in the last three decades have been met with mixed results. Although most healthcare providers working in maternity settings know about the partograph, it is frequently used retrospectively for recording purposes instead of providing prospective support for clinical decision making. Possible reasons for these shortcomings include difficulties in its use and interpretation.^26^, ^27^ Our findings suggest that the poor predictive performance of the partograph – and the consequent effect in supporting effective decision making – could contribute to the lack of interest in using the tool prospectively.

As countries navigate through the obstetric transition,^28^ a marked trend towards the medicalisation of labour and childbirth is observed. Several determinants of the medicalisation of labour and childbirth are at play, including models of care based on the notion that a normal labour abides by the ‘one‐centimetre per hour’ rule. This notion has been embedded in generations of healthcare providers across the world, and the implicit or explicit influence of this notion in obstetric and midwifery culture cannot be over‐emphasised; however, as suggested by our findings, a cervical dilatation rate of ‘one‐centimetre per hour’ may be unrealistically fast for a substantial proportion of women in labour. The mismatch between the unrealistic expectations of healthcare providers and the physiology of labour may give rise to the constructed ‘need’ for an intervention in a natural process that could otherwise be slower than currently expected but end well and naturally. The poor accuracy of the tool means on one hand that a high proportion of women would receive an intervention without a valid justification, and on the other hand women at risk would not be recognised in time to avoid the adverse outcome. The excessive use of interventions may also contribute to adverse outcomes. For example, the augmentation of labour is a well‐established risk factor for fetal distress; unnecessary augmentation of labour, prompted by the ‘one‐centimetre per hour’ rule, may be harmful, particularly in settings with limited capacity for providing appropriate, intermittent fetal monitoring. Another potential adverse effect of the above mismatch is increased tension, anxiety, and frustration among the staff, which could be a contributing factor to disrespect, abuse, and mistreatment of women during labour and childbirth. Allowing an increase in the average duration of labour in health facilities has a direct impact on the occupancy rate of labour‐ward beds, however, which could further complicate the shortage of hospital beds and overcrowding of health facilities. Reducing the number of interventions during labour could reduce staff workload. Research to determine the short‐ and long‐term consequences of a less invasive intrapartum care model at the individual and at the health‐systems level is warranted.

The poor performance of the partograph and the customised labour curves may not be a surprising finding. The rationale for using the partograph for preventing labour problems goes back several decades, when it was introduced for the timely referral from peripheral health facilities to prevent the complications of obstructed labour.^9^ Fetal and early neonatal outcomes are much more likely to be impacted by events that are not related to the cervical dilatation rate, such as placental abruption, cord compression, cord prolapse, meconium aspiration, and intrauterine growth restriction, among many other reasons. In South Africa, for example, only 6% of fetal and early neonatal deaths were associated with prolonged labour;^29^ however, we should not overlook the finding that slower labours compared with faster labours (in different percentiles) tended to be associated with a mild increase in the risk of adverse outcomes. Nevertheless, this association alone can hardly provide a basis for a reliable classification tool because of the excessive number of false positives. For example, nearly half of the study population crossed the alert line, making the policy of transferring women who crossed the alert line to referral hospitals impractical. In this context, and given the limitations of static, paper‐based diagnostic tools, the development and testing of more sophisticated, dynamic, easy‐to‐use tools for improved risk classification during labour is a priority. A cluster‐randomised trial, comparing a static paper‐based partograph with a dynamic, multivariable prediction model would be ideal research to be carried out next.

## Conclusion

Our findings suggest that the validity of a partograph alert line based on the ‘one‐centimetre per hour’ rule should be re‐evaluated. Labour is an extremely variable phenomenon, and emphasis should be given to individualised, supportive, person‐centered care during labour and childbirth.

### Disclosure of interests

None declared. Completed disclosure of interests form available to view online as supporting information.

### Contribution to authorship

JPS, OTO, and AMG developed the research protocol, with input from members of the project steering committee and advisory group, Nigeria and Uganda research teams, data science team, and the project coordination and support team. The analysis plan was developed by JPS with input from OTO. Data analysis was carried out by RR, FBJ, LOC, and JPS. JPS drafted this article, with substantial contributions from OTO, BF, KM, RR, FBJ, LOC, DA, and AMG. All authors reviewed the draft manuscript for intellectual content and approved the final version for publication.

### Details of ethics approval

Scientific and technical approval was obtained from the Review Panel on Research Projects (RP2) of UNDP/UNFPA/UNICEF/WHO/World Bank Special Program of Research, Development and Research Training in Human Reproduction (HRP). Ethical approval was obtained from the World Health Organization Ethical Review Committee (protocol A65879, approval date 25 August 2014), the Makerere University School of Health Sciences Research and Ethics Committee, Uganda (protocol #SHSREC REF 2014‐058), University of Ibadan/University College Hospital Ethics Committee (UI/EC/14/0223), Federal Capital Territory Health Research Ethics Committee, Nigeria (protocol FHREC/2014/01/42/27‐08‐14), and Ondo State Government Ministry of Health Research Ethics Review Committee, Nigeria (AD 4693/160).

## Supporting information


**Figure S1.** The analysis flowchart.

**Table S1.** Calculation of diagnostic accuracy statistics.
**Table S2.** Equivalence of diagnostic accuracy statistics.
**Table S3.** Suggested interpretation of diagnostic accuracy statistics.
**Table S4.** Distribution of the study population according to obstetric groups and the frequency of severe adverse birth outcomes.
**Table S5.** Diagnostic accuracy of customized labour curves by percentiles.
**Table S6.** Diagnostic accuracy of other predictors included in the partograph.
**Table S7**. Definitions of the predictors included in the Table S6 and Figure S1.

**Box S1.** Sample Size calculation.
**Box S2.** The J statistic in the ROC space

**Video S1.** All women and perinatal outcome
