# Supplementary figures and images for: Cervical dilatation over time is a poor predictor of severe adverse birth outcomes: a diagnostic accuracy study
Source: BJOG. 2018 Apr 17;125(8):991–1000. doi: 10.1111/1471-0528.15205 (PMC6032950; doi:10.1111/1471-0528.15205)

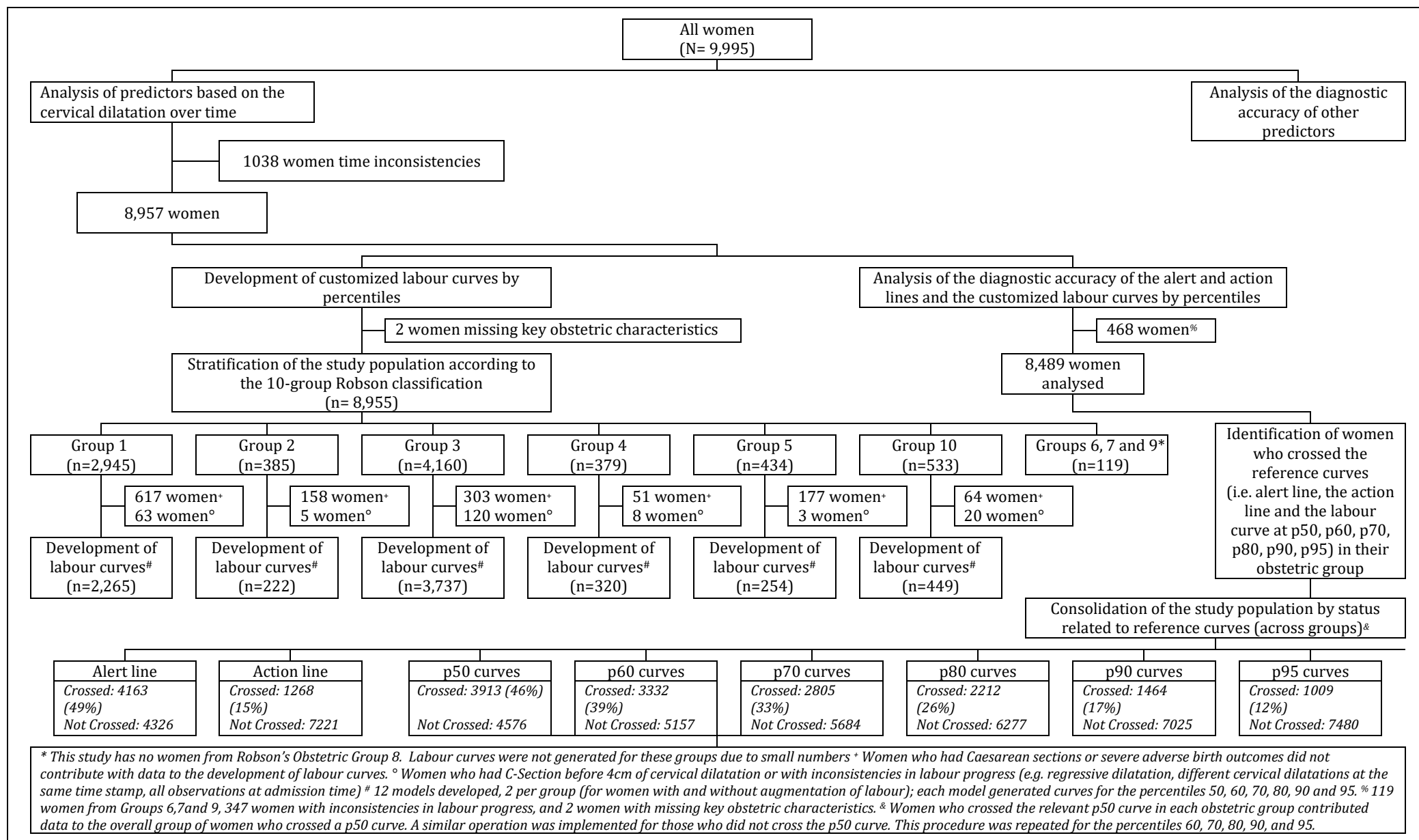

**Figure S1.** The analysis flowchart

Supplement: Supplementary file 1 — Figure S1. The analysis flowchart. [file BJO-125-991-s001.pdf]
