# Supplementary material for: Cervical dilatation over time is a poor predictor of severe adverse birth outcomes: a diagnostic accuracy study
Source: BJOG. 2018 Apr 17;125(8):991–1000. doi: 10.1111/1471-0528.15205 (PMC6032950; doi:10.1111/1471-0528.15205)
Supplement: Supplementary file 2 — Table S1. Calculation of diagnostic accuracy statistics. Table S2. Equivalence of diagnostic accuracy statistics. Table S3. Suggested interpretation of diagnostic accuracy statistics. Table S4. Distribution of the study population according to obstetric groups and the frequency of severe adverse birth outcomes. Table S5. Diagnostic accuracy of customized labour curves by percentiles. Table S6. Diagnostic accuracy of other predictors included in the partograph. Table S7. Definitions of the predictors included in the Table S6 and Figure S1. [file BJO-125-991-s002.pdf]

Table S1. Calculation of diagnostic accuracy statistics

Clinical Signs		Severe adverse birth outcomes		Sensitivity	Specificity	Positive Likelihood Ratio	Negative Likelihood Ratio	Diagnostic Odds Ratio	J statistic
		Present	Absent						
Predictor	Positive	TP	FP	TP / (TP+FN)	TN / (FP+TN)	Sens / (1-Sp)	(1-Sens) / Sp	(TP * TN) / (FP * FN)	Sens+ Sp - 1
	Negative	FN	TN						

Table S2. Equivalence of diagnostic accuracy statistics

Sensitivity	Specificity	Positive Likelihood Ratio	Negative Likelihood Ratio	Diagnostic Odds Ratio	J statistic [Youden's Index]	AUROCC
50%	50%	1.00	1.00	1.00	0.00	0.500
60.0%	60.0%	1.50	0.67	2.25	0.20	0.600
70.0%	70.0%	2.33	0.43	5.44	0.40	0.700
80.0%	80.0%	4.00	0.25	16.00	0.60	0.800
90.0%	90.0%	9.00	0.11	81.00	0.80	0.900
90.9%	90.9%	9.99	0.10	99.78	0.82	0.909
95.0%	95.0%	19.00	0.05	361.00	0.90	0.950
99.99%	99.99%	9999.00	0.00	99980001.00	1.00	1.000

AUROCC: Area Under the Receiver Operator Characteristic Curve
Positive Likelihood Ratio: The probability of a person who has the disease testing positive divided by the probability of a person who does not have the disease testing positive
Negative Likelihood Ratio: The probability of a person who has the disease testing negative divided by the probability of a person who does not have the disease testing negative

Table S3. Suggested interpretation of diagnostic accuracy statistics[16,18,19,30]

Interpretation	Positive Likelihood Ratio	Negative Likelihood Ratio	Diagnostic Odds Ratio	J statistic [Youden's Index]	AUROCC
Non-discrimination	1.00	1.00	1.00	0.00	0.500
Poor	1.01-1.50	0.99-0.67	1.01-2.25	0.01-0.20	0.501-0.600
Intermediate	1.51-9.99	0.66-0.11	2.26-99.99	0.21-0.79	0.601-0.899
Excellent	≥10.00	≤0.10	≥100.00	≥0.80	≥0.900

AUROCC: Area Under the Receiver Operator Characteristic Curve
Positive Likelihood Ratio: The probability of a person who has the disease testing positive divided by the probability of a person who does not have the disease testing positive
Negative Likelihood Ratio: The probability of a person who has the disease testing negative divided by the probability of a person who does not have the disease testing negative

Table S4. Distribution of the study population according to obstetric groups and the frequency of severe adverse birth outcomes

	n	%
Obstetric groups (N=8,955)		
Group 1 (nulliparous women with singleton cephalic pregnancy, 37 weeks gestation or more in spontaneous labour)	2,945	32.9%
Group 2 (nulliparous women with singleton cephalic pregnancy, 37 weeks gestation or more, induced onset of labour)	385	4.3%
Group 3 (multiparous women without previous caesarean section, with singleton cephalic pregnancy, 37 weeks gestation or more in spontaneous labour)	4,160	46.5%
Group 4 (multiparous women without previous caesarean section, with singleton cephalic pregnancy, 37 weeks gestation or more, induced onset of labour)	379	4.3%
Group 5 (All multiparous women with at least one previous caesarean section, singleton cephalic pregnancy, 37 weeks gestation or more)	434	4.8%
Group 10 (All women with singleton cephalic preterm pregnancy (less than 37 weeks gestation at childbirth)	533	6.0%
Groups 6, 7 and 9 (All other women with non-cephalic presentations, excluding multiple pregnancies)	119	1.3%
Severe adverse birth outcomes (N=9995)		
Apgar score 5’ < 6	143	1.4%
Neonatal cardiopulmonary resuscitation	71	0.7%
Early neonatal death	57	0.6%
Neonatal use of anticonvulsants	36	0.4%
Stillbirths	23	0.2%
C-Section due to suspected uterine rupture	11	0.1%
Severe maternal outcome associated with dystocia**	10	0.1%
Uterine rupture	7	0.1%
**Any of the above**	**223**	**2.2%**

*1,038 women with time inconsistencies and two women with missing information on the key obstetric characteristics were not classified according to the 10-group Robson classification. Group X is composed of women originated from the Robson’s Obstetric Groups 6, 7 and 9. This study has no women from Robson’s Obstetric Group 8.
** Includes 6 maternal deaths and 4 maternal near-miss cases (survivors of organ dysfunction)

Table S5. Diagnostic accuracy of customized labour curves by percentiles

		Severe adverse birth outcomes		Sensitivity (95% CI)	Specificity (95% CI)	Positive Likelihood Ratio (95% CI)	Negative Likelihood Ratio (95% CI)	Diagn ostic Odds Ratio (95% CI)	J statistic [Youden's Index] (95% CI)
		Present	Absent						
p50	Crossed	103	3810	53.1% (46.1-60.1)	54.1% (53.0-55.1)	1.16 (1.01-1.32)	0.87 (0.75-1.01)	1.33 (1.00-1.77)	7.2% (0.1-14.3)
	Not crossed	91	4485						
p60	Crossed	88	3244	45.4% (38.4-52.4)	60.9% (59.8-61.9)	1.16 (0.99-1.36)	0.90 (0.79-1.02)	1.29 (0.97-1.72)	6.3% (-0.8 – 13.3)
	Not crossed	106	5051						
p70	Crossed	81	2724	41.8% (34.8-48.7)	67.2% (66.2-68.2)	1.27 (1.07-1.51)	0.87 (0.77-0.98)	1.47 (1.10-1.96)	8.9% (1.9 – 15.9)
	Not crossed	113	5571						
p80	Crossed	69	2143	35.6% (28.8-42.3)	74.2% (73.2-75.1)	1.38 (1.14-1.67)	0.87 (0.78-0.97)	1.59 (1.18-2.14)	9.7% (2.9-16.5)
	Not crossed	125	6152						
p90	Crossed	46	1418	23.7% (17.7-29.7)	82.9% (82.1-83.7)	1.39 (1.07-1.79)	0.92 (0.85-1.00)	1.51 (1.08-2.11)	6.6% (0.6 – 12.7)
	Not crossed	148	6877						
p95	Crossed	34	975	17.5% (12.2-22.9)	88.2% (87.6-88.9)	1.49 (1.09-2.04)	0.93 (87.5-1.00)	1.60 (1.10-2.32)	5.8% (0.4 – 11.2)
	Not crossed	160	7320						

Table S6. Diagnostic accuracy of other predictors included in the partograph*

		Severe adverse birth outcomes		Sensitivity	Specificity	Positive Likelihood Ratio	Negative Likelihood Ratio	Diagnostic Odds Ratio	J statistic [Youden's Index]
		**Present**	**Absent**	**(95%CI)**	**(95%CI)**	**(95%CI)**	**(95%CI)**	**(95%CI)**	**(95%CI)**
Meconium	Present	62	1220	28.4% (22.5-34.4)	87.1% (86.4-87.7)	2.2 (1.77-2.73)	0.82 (0.76-0.89)	2.67 (1.98-3.61)	15.5% (9.5-21.5)
	Absent	156	8203						
Significant Caput Succedaneum	Present	31	390	14.2% (9.5-18.8)	95.9% (95.4-96.3)	3.41 (2.43-4.79)	0.9 (0.85-0.94)	3.81 (2.57-5.64)	10.0% (5.4-14.6)
	Absent	188	9011						
Abnormal maternal temperature	Present	24	362	11.9% (7.5-16.4)	95.7% (95.2-96.1)	2.76 (1.87-4.07)	0.92 (0.87-0.97)	3 (1.93-4.65)	7.6% (3.1-12.1)
	Absent	177	8003						
Significant moulding	Present	22	327	10.0% (6.0-14.0)	96.5% (96.2-96.9)	2.88 (1.91-4.33)	0.93 (0.89-0.97)	3.08 (1.96-4.86)	6.5% (2.5-10.5)
	Absent	198	9075						
Fetal movements not observed	Present	55	1744	25.0% (19.3-30.7)	81.4% (80.6-82.2)	1.35 (1.07-1.70)	0.92 (0.85-0.99)	1.46 (1.07-1.99)	6.4% (0.7-12.2)
	Absent	165	7649						
Abnormal fetal heart rate	Present	16	190	7.3% (3.8-10.7)	98.0% (97.7-98.3)	3.63 (2.22-5.93)	0.95 (0.91-0.98)	3.83 (2.26-6.50)	5.3% (1.8 – 8.7)
	Absent	204	9281						
Slow descent	Present	40	1243	22.0% (16.0-28.0)	83.2% (82.4-84.1)	1.31 (0.99-1.73)	0.94 (0.87-1.01)	1.4 (0.98-2.00)	5.2% (-0.9 – 11.3)

	Absent	142	6176						
Suboptimal duration of uterine contractions	Present	39	1407	18.7% (13.4-23.9)	83.9% (83.1-84.7)	1.16 (0.87-1.55)	0.97 (0.91-1.03)	1.2 (0.84-1.70)	2.6% (-2.8 – 7.9)
	Absent	170	7341						
Abnormal systolic blood pressure	Present	12	318	5.6% (2.5-8.7)	96.4% (96.0-96.8)	1.58 (0.90-2.76)	0.98 (0.95-1.01)	1.61 (0.89-2.92)	2.1% (-1.1 – 5.2)
	Absent	201	8595						
Abnormal position of fetal head	Present	4	66	1.9% (0.1-3.8)	99.3% (99.1-99.5)	2.71 (0.99-7.63)	0.99 (0.97-1.00)	2.74 (0.99-7.60)	1.2% (-0.7 – 3.1)
	Absent	203	9188						
Abnormal maternal heart rate	Present	6	153	2.8% (0.6-5.1)	98.3% (98.0-98.6)	1.66 (0.74-3.72)	0.99 (0.97-1.01)	1.68 (0.73-3.85)	1.1% (-1.1 – 3.4)
	Absent	206	8835						
Suboptimal number of contractions	Present	32	1269	15.3% (10.4-20.2)	85.5% (84.8-86.2)	1.06 (0.76-1.46)	0.99 (0.93-1.05)	1.07 (0.73-1.56)	0.8% (-4.1 – 5.7%)
	Absent	177	7479						
Abnormal diastolic blood pressure	Present	2	110	0.9% (0.0-2.2)	98.8% (98.5-99.0)	0.76 (0.189-3.06)	1 (0.99-1.016)	0.76 (0.19-3.09)	-0.3% (-1.6 – 1.0)
	Absent	211	8809						

*Table S7 presents the definitions used for each predictor.

Table S7. Definitions of the predictors included in the Table S6 and Figure S1

Predictor	Codification rule
Meconium	If the membranes status is ruptured with stale meconium or with fresh meconium, then meconium is present
Significant Caput Succedaneum	If caput succedaneum is moderate or severe, then the caput succedaneum is significant
Abnormal maternal temperature	If maternal temperature>37.5 or maternal temperature<35.5 then the maternal temperature is abnormal
Significant moulding	If moulding is second or third degree, then the moulding is significant
Fetal movements not observed	If no movement is observed or felt
Abnormal fetal heart rate	If fetal heart rate <110 OR fetal heart rate>160 then the fetal heart rate is abnormal
Slow descent	If the descent is slower than 90% of women in the database, then the descent is slow. Threshold established through a multi-state Markov model.
Suboptimal duration of uterine contractions	If duration of uterine contractions > 60s OR if labour is slow and duration of uterine contractions < 40s, then the duration of uterine contractions is suboptimal
Abnormal systolic blood pressure	If systolic blood pressure>159 or systolic blood pressure<85, then the systolic blood pressure is abnormal
Abnormal position of fetal head	If in the last vaginal examination, position of fetal head is occipito-transverse or occipito-posterior or a position other than occipito-anterior, then the position of fetal head is abnormal
Abnormal maternal heart rate	If maternal heart rate>119 or maternal heart rate<60 then the maternal heart rate is abnormal
Suboptimal number of contractions	If number of uterine contractions in 10 minutes > 4 OR if labour is slow and number of uterine contractions in 10 minutes < 3 then the number of contractions is suboptimal
Abnormal diastolic blood pressure	If diastolic blood pressure>105 then the diastolic blood pressure is abnormal
