# Supplementary material for: Cervical dilatation over time is a poor predictor of severe adverse birth outcomes: a diagnostic accuracy study
Source: BJOG. 2018 Apr 17;125(8):991–1000. doi: 10.1111/1471-0528.15205 (PMC6032950; doi:10.1111/1471-0528.15205)
Supplement: Supplementary file 3 — Box S1. Sample Size calculation. Box S2. The J statistic in the ROC space [file BJO-125-991-s003.pdf]

Box S1. Sample Size calculation

The required sample size for this project considering its main objectives, which go beyond this specific analysis. In order to achieve the main objective of this project, a total of 7,812 women in early labour are needed. The sample size calculation was based on the number of candidate predictors (N = 20 (maximum number)), the minimum number of outcomes per predictor considered for model development and validation (M = 15; 10 in the training set and 5 in the validation set); I = incidence of the main outcome of interest (I = 4.8%) and a margin of error (ME = 25%, also accounting for the clustering effect).

$$\textbf{Sample size} = ((N \times M)/I) \times (1 + ME) = ((20 \times 15)/0.048) \times (1 + 0.25) = 7,812$$

The incidence of the main outcome of interest was based on data derived from the WHO Multicountry Survey on Maternal and Newborn Health (WHO MCS) in Nigeria and Uganda (13).

The number of health facilities was determined based on the average annual number of births of district/secondary level hospitals that participated in the WHO Multicountry Survey on Maternal and Newborn Health for Nigeria and Uganda and a recent census carried out among candidate health facilities. Considering a 6-month data collection period and that only 50% of the women are in early labour, eligible and willing to participate a total of eight health facilities (4 per country) will take part of this study (participating hospitals are expected to recruit 1,000 women on average).

Box S2. The J statistic in the ROC space

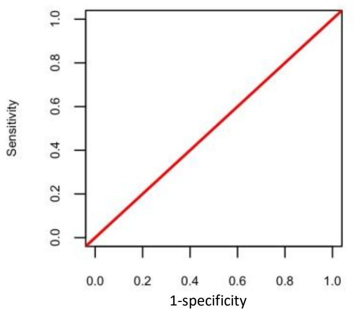

In the ROC space, the slope of the non-discrimination line is given by:

$m = (Y - Y_0)/(X - X_0)$
$Y = 1.0$
$Y_0 = 0$
$X = 1.0$
$X_0 = 0$
$m = (Y-0)/(X-0)$
$m = Y/X$
$m = 1$

$Y/X = 1$

In the non-discrimination line, X=Y

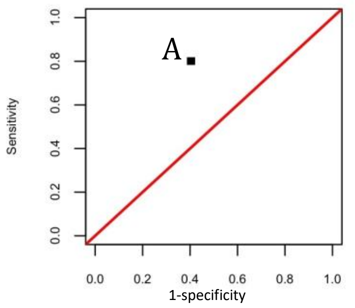

The point A, represents the performance of a diagnostic test. The point A has the coordinates ((1 – specificity), sensitivity) or (x,y);

x= 1 – specificity
y= sensitivity

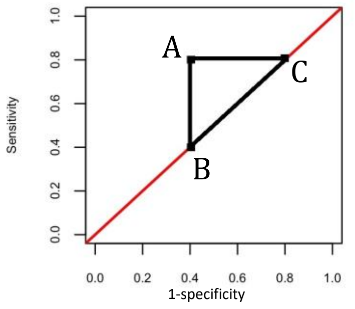

Consider the points A, B, C;

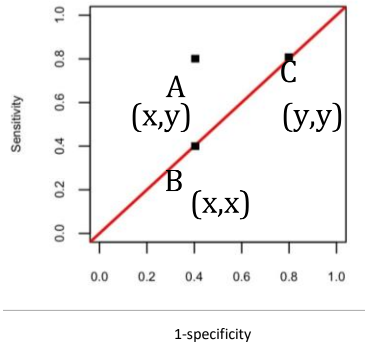

And their coordinates;

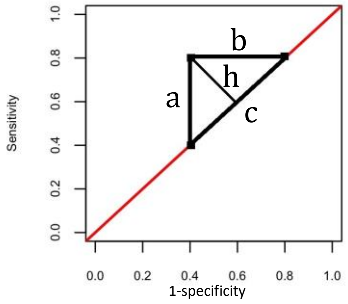

$a = y - x$

$b = x - y$

$a^2 = b^2$

$c^2 = a^2 + b^2$

$c^2 = 2a^2$

$a^2 = h^2 + \left(\frac{c}{2}\right)^2$

$h^2 = a^2 - \left(\frac{c}{2}\right)^2$

$h^2 = a^2 - \left(\frac{c^2}{4}\right)$

$h^2 = a^2 - \left(\frac{2a^2}{4}\right)$

$h^2 = a^2 - \left(\frac{a^2}{2}\right)$

$h^2 = \frac{a^2}{2}$

$h = \sqrt{\frac{a^2}{2}}$

$h = \sqrt{\frac{(sensitivity - (1 - specificity))^2}{2}}$

$h = \sqrt{\frac{(sensitivity + specificity - 1)^2}{2}}$

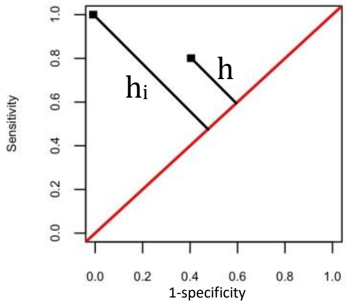

h_i is equivalent to the half of the diagonal d of a 1.0 X 1.0 square

$d^2 = 1^2 + 1^2$

$d^2 = 2$

$d = \sqrt{2}$

$h_i = \frac{\sqrt{2}}{2}$

Considering that h is an expression of diagnostic performance of any given test and h_i is an expression of the diagnostic performance of the ideal test, the ratio between h and h_i expresses the proportion of ideal diagnostic performance achieved by a given test (h/h_i).

$h/h_i = \frac{\sqrt{\frac{(sensitivity + specificity - 1)^2}{2}}}{\frac{\sqrt{2}}{2}}$

$h/h_i = sensitivity + specificity - 1$

The proportion h/h_i, when written in the form $(sensitivity + specificity - 1)$ is seen to be equivalent to the J statistic (Youden index).
